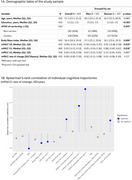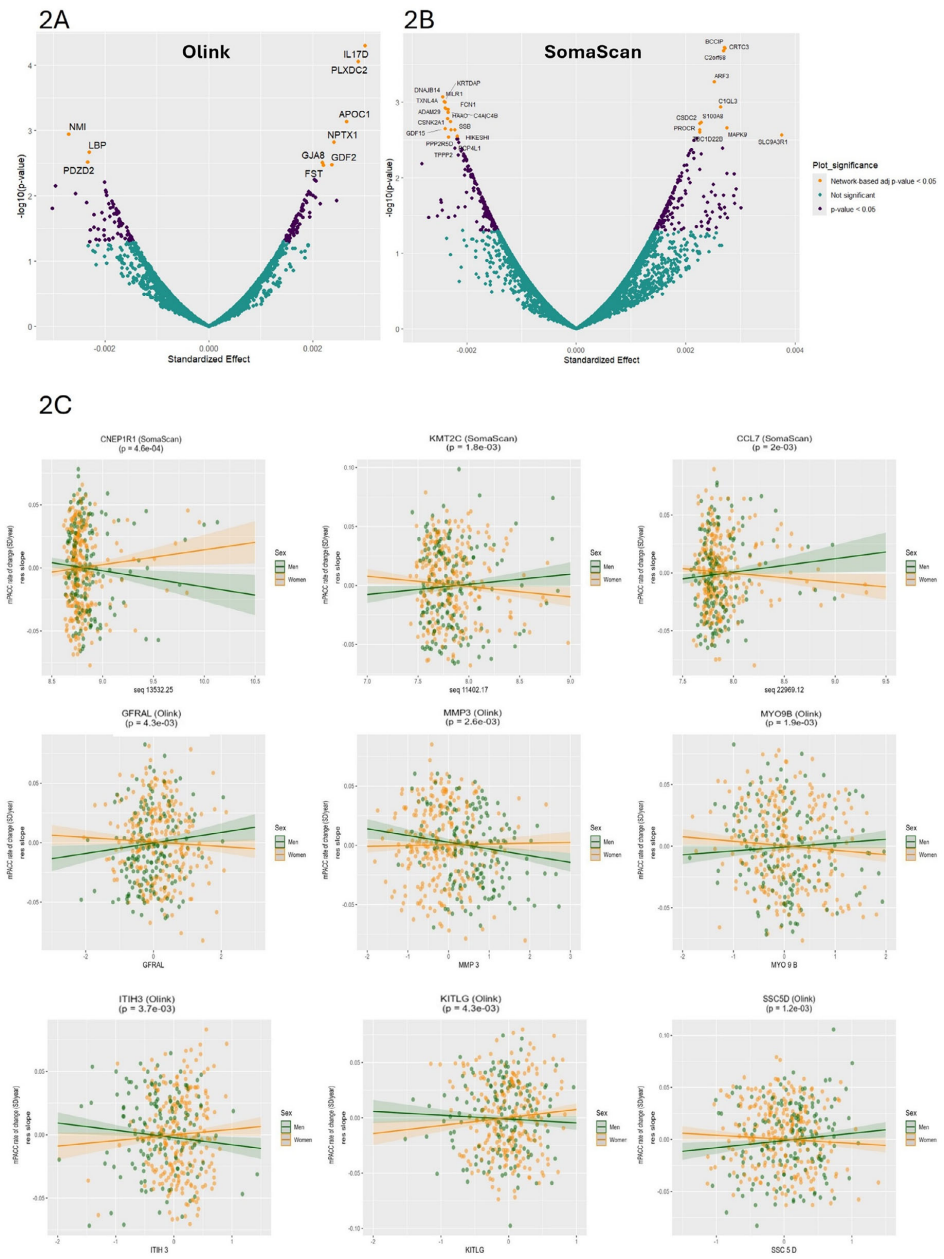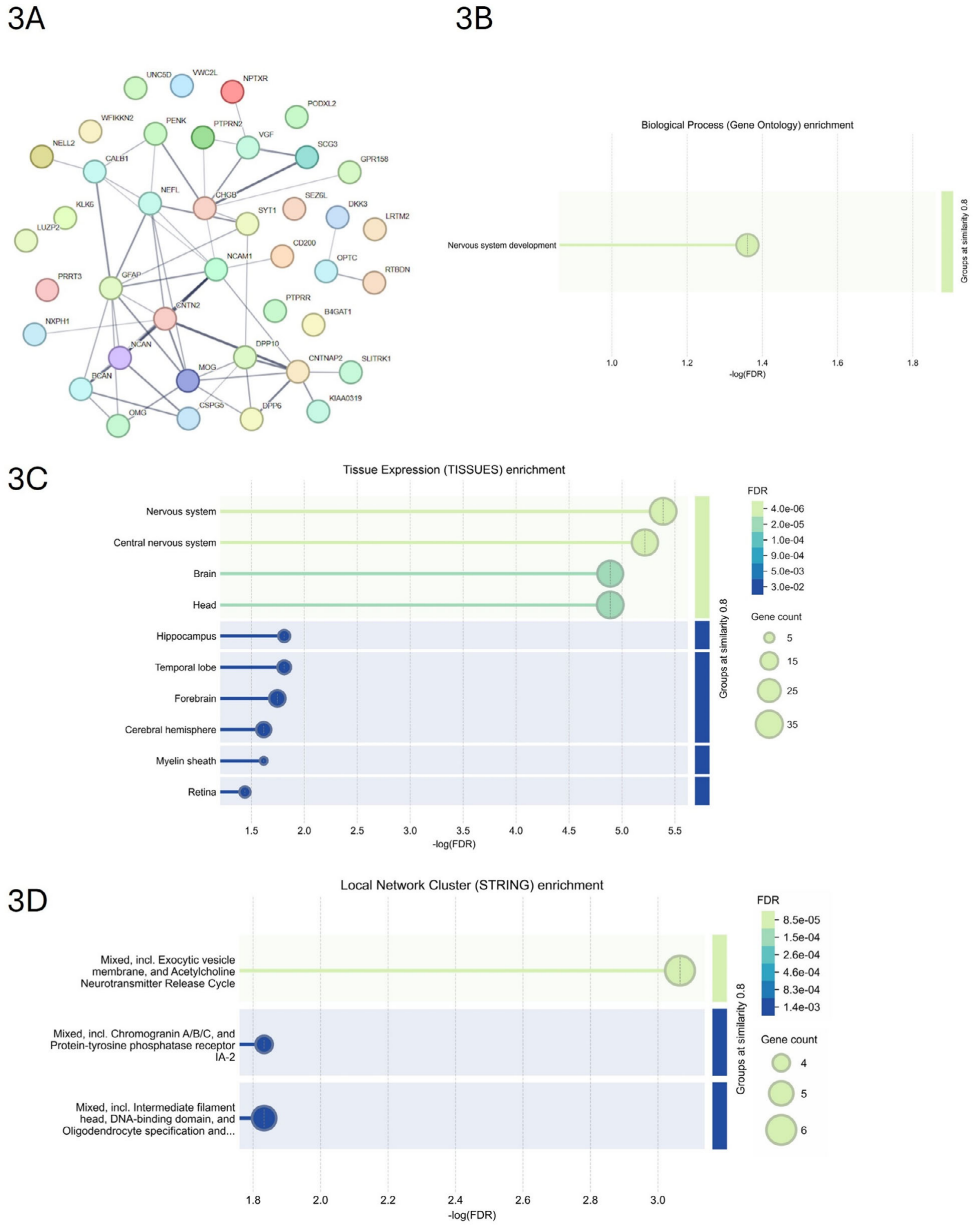# Predicting cognitive trajectories in asymptomatic individuals at‐risk of AD: insights from a multi‐platform plasma proteomics study

**DOI:** 10.1002/alz70856_099234

**Published:** 2025-12-24

**Authors:** Federica Anastasi, Armand González Escalante, Pol Segura‐Retana, Paula Ortiz‐Romero, Gonzalo Sánchez‐Benavides, David López‐Martos, Oriol Grau‐Rivera, Carolina Minguillón, Marta del Campo, Natalia Vilor‐Tejedor, Marc Suárez‐Calvet

**Affiliations:** ^1^ Barcelonaβeta Brain Research Center (BBRC), Pasqual Maragall Foundation, Barcelona, Spain; ^2^ Hospital del Mar Research Institute (IMIM), Barcelona, Spain; ^3^ Centre for Genomic Regulation (CRG), Barcelona Institute of Science and Technology (BIST), Barcelona, Spain; ^4^ Universitat Pompeu Fabra, Barcelona, Spain; ^5^ Centro de Investigación Biomédica en Red de Fragilidad y Envejecimiento Saludable (CIBERFES), Instituto de Salud Carlos III, Madrid, Spain; ^6^ Servei de Neurologia, Hospital del Mar, Barcelona, Spain; ^7^ Instituto de Salud Carlos III, Madrid, Spain; ^8^ Hospital del Mar Research Institute, Barcelona, Spain; ^9^ Radboud University Medical Center, Nijmegen, Netherlands

## Abstract

**Background:**

Plasma biomarkers enabling early detection of Alzheimer's disease (AD) biological hallmarks are now available. Yet, predicting cognitive decline in at‐risk individuals remains challenging due to the high variability of cognitive outcomes and lack of reliable prognostic markers. This study investigated baseline plasma proteins associated with 7.5‐year cognitive trajectories in asymptomatic individuals at‐risk of AD, with a focus on sex‐specific proteins.

**Method:**

We included 410 cognitively unimpaired individuals (baseline median age: 57.1 years; *APOE*‐ɛ4 carriers: 56%; women: 60%, Figure 1A). Cognitive trajectories (slopes) were derived from a linear mixed‐effects model using three cognitive assessments (modified Preclinical Alzheimer Cognitive Composite, mPACC), corrected by age. Linear associations between cognitive trajectories and baseline plasma proteins (∼3K Olink Explore, ∼7K SomaScan v4.1) were assessed. Sex‐interactions were tested to identify sex‐specific predictors of cognitive changes. Models were adjusted for age, sex and average total proteome. Participants under the 25th percentile of mPACC trajectories were classified as decliners. Weighted correlation network analysis (WGCNA) was conducted to identify co‐expressed protein modules, which were tested using logistic regression to predict decliner status. Module enrichment was assessed using STRING (v.12.0, FDR<0.05) using Olink and SomaScan identified proteins as reference datasets.

**Result:**

Cognitive trajectories (range: ‐0.59,0.71 SD/10 years) were negatively correlated with age, body mass index, and CSF‐NfL, and positively correlated with FDG‐PET, mPACC follow‐ups and education (Figure 1B). After applying a Bonferroni‐like correction for network number, 10 Olink and 26 SomaScan proteins were significantly associated with cognitive trajectories, with IL17D and NHERF1 (encoded by *SLC9A3R1*) having the biggest effect size for Olink and SomaScan, respectively (Figure 2A‐B). Ten significant sex‐protein interactions predicting cognitive change were also identified (Figure 2C). WGCNA revealed 9 protein modules for Olink and 17 for SomaScan. Logistic regression analyses found one Olink module significantly associated with the decliner status (*p* = 0.025). This module, comprising 40 proteins, was enriched in CNS tissues and biological processes related to nervous system development (e.g., NEFL, GFAP, NCAM1, NPTXR; Figure 3A‐3D).

**Conclusion:**

Plasma proteins can predict differential cognitive trajectories in asymptomatic individuals at‐risk for AD, demonstrating their potential as prognostic markers. Additionally, sex‐specific associations highlight the importance of personalized approaches in risk stratification.